# PRMT5 Inhibitors Regulate DNA Damage Repair Pathways in Cancer Cells and Improve Response to PARP Inhibition and Chemotherapies

**DOI:** 10.1158/2767-9764.CRC-23-0070

**Published:** 2023-11-06

**Authors:** Jack Carter, Michael Hulse, Monisha Sivakumar, Jessica Burtell, Venkat Thodima, Min Wang, Anjana Agarwal, Komali Vykuntam, Jacob Spruance, Neha Bhagwat, Joseph Rager, Bruce Ruggeri, Peggy Scherle, Koichi Ito

**Affiliations:** 1 Prelude Therapeutics Incorporated, Wilmington, Delaware.

## Abstract

Expression of protein arginine methyltransferase 5 (PRMT5) is highly positively correlated to DNA damage repair (DDR) and DNA replication pathway genes in many types of cancer cells, including ovarian and breast cancer. In the current study, we investigated whether pharmacologic inhibition of PRMT5 downregulates DDR/DNA replication pathway genes and sensitizes cancer cells to chemotherapy and PARP inhibition. Potent and selective PRMT5 inhibitors significantly downregulate expression of multiple DDR and DNA replication genes in cancer cells. Mechanistically, PRMT5 inhibition reduces the presence of PRMT5 and H4R3me2s on promoter regions of DDR genes such as *BRCA1/2*, *RAD51*, and *ATM*. PRMT5 inhibition also promotes global alternative splicing changes. Our data suggest that PRMT5 inhibition regulates expression of *FANCA*, *PNKP*, and *ATM* by promoting exon skipping and intron retention. Combining C220 or PRT543 with olaparib or chemotherapeutic agents such as cisplatin demonstrates a potent synergistic interaction in breast and ovarian cancer cells *in vitro*. Moreover, combination of PRT543 with olaparib effectively inhibits the growth of patient-derived breast and ovarian cancer xenografts. Furthermore, PRT543 treatment significantly inhibits growth of olaparib-resistant tumors *in vivo*. These studies reveal a novel mechanism of PRMT5 inhibition and suggest beneficial combinatorial effects with other therapies, particularly in patients with tumors that are resistant to therapies dependent on DNA damage as their mechanism of action.

**Significance:**

Patients with advanced cancers frequently develop resistance to chemotherapy or PARP inhibitors mainly due to circumvention and/or restoration of the inactivated DDR pathway genes. We demonstrate that inhibition of PRMT5 significantly downregulates a broad range of the DDR and DNA replication pathway genes. PRMT5 inhibitors combined with chemotherapy or PARP inhibitors demonstrate synergistic suppression of cancer cell proliferation and growth in breast and ovarian tumor models, including PARP inhibitor–resistant tumors.

## Introduction

Genetic defects in DNA damage repair (DDR) pathways are clinically proven to induce synthetic lethality with chemotherapy and PARP inhibitors. However, patients with advanced disease develop resistance to these therapies by restoring or circumventing these pathways (1, 2). Rewiring of the epigenome is a fundamental change in many cancers that globally impacts gene expression, a finding that has led to the development of epigenetic-targeted therapies (3, 4). Protein arginine methyltransferase 5 (PRMT5) has emerged as an important epigenetic regulator with essential roles in promoting tumor growth and survival. PRMT5 expression is ubiquitous and its overexpression in cancers correlates with disease progression and worse prognosis (5–8). Accordingly, PRMT5 inhibition is being explored as a cancer therapy and several PRMT5 inhibitors have been tested in clinical trials (9–12). Thus, elucidating molecular changes and sensitivities induced by PRMT5 inhibition can inform potential opportunities in the clinic, such as biomarker selection and combination therapies.

As the predominant type II PRMT, PRMT5 catalyzes the symmetric dimethylation of arginine (sDMA) residues on histones and non-histone protein substrates to regulate gene expression (11, 12). PRMT5 methyltransferase activity is regulated predominantly by the cofactor MEP50 (12, 13), though independent mechanisms have been reported (14). Functioning as an epigenetic writer through arginine methylation of histones, PRMT5 alters gene expression, while its modification of non-histone proteins disrupts RNA splicing and processing, cell signaling, and ribosome biogenesis (8, 15, 16). Indeed, PRMT5 is a major regulator of the spliceosome which globally impacts mRNA splicing (17, 18). Ablation of PRMT5 induces alternative splicing (AS) in pre-mRNAs which can induce nonsense-mediated decay and disrupt protein expression or function (18–20). Interestingly, spliceosome-mutated cancers may represent a synthetic lethal vulnerability to PRMT5-targeting therapies (17).

Maintenance of DNA double-strand breaks, and thereby genomic integrity, are crucial for cell survival. The primary DNA repair mechanisms involve homologous recombination (HR) and non-homologous end joining pathways which rely on several essential DNA repair proteins (21). Consequently, cancer cells with genetic defects in DDR pathways are often vulnerable to DNA damage (21, 22). This vulnerability has been clinically validated with the success of PARP inhibitors in patients with ovarian, breast, prostate, and pancreatic cancers deficient in HR factors homologous recombination deficiency (HRD), such as those harboring mutations in *BRCA1* or *BRCA2* (22–25). Furthermore, HRD status is predictive of response to DNA damage–inducing chemotherapy (26, 27). Conversely, strengthened DNA repair mechanisms, either through circumvention and/or restoration of inactivated DDR genes, can lead to resistance to such agents (28, 29). Previous works by our and other groups have associated PRMT5 with the regulation of DDR genes in blood and prostate cancers (14, 16), although the precise mechanisms remain unclear and therapeutic opportunities continue to be explored. Consistent with its epigenetic function, PRMT5 induces symmetric dimethylation of H4R3 (H4R3me2s) to activate DDR gene expression in irradiated prostate cancer cells (14). Depletion or inhibition of PRMT5 reduces global H3R2me1 basally enriched at several Fanconi anemia (FA) gene promoters, reducing their expression in glioblastoma (30). Interestingly, PRMT5 is also associated with enriched AS of genes in DDR pathways in blood and brain cancers (19, 20, 31, 32), suggesting PRMT5 may act as a major regulator of DDR genes through several layers of mechanism.

Preclinical evidence suggests PRMT5 regulation of DDR pathways may be a therapeutic opportunity. PRMT5 loss improves sensitivity of cancer cell lines to radiation (14) and interstrand cross-linking agents (30). Importantly, preclinical PRMT5 inhibitors increase DNA damage and improve the sensitivity of leukemia cells to PARP or ATM inhibitors *in vitro* (20). Thus, we sought to evaluate therapeutic opportunities for our PRMT5 inhibitors (C220 and PRT543) in ovarian and breast cancers, where PARP inhibitors are clinically approved in patients with HRD tumors (24, 25). We show here evidence that PRMT5 inhibitors suppress DDR gene expression through epigenetic and AS mechanisms and enhance the sensitivity of ovarian and breast cancers to chemotherapy and PARP inhibitors *in vitro* and *in vivo*. Our work suggests PRMT5 inhibitors could be used in combination with chemotherapy or PARP inhibitors to treat ovarian or breast cancers, including in genetically proficient tumors through the induction of chemically induced synthetic lethality.

## Materials and Methods

### Cell Culture, Cell Line Generation, Reagents, and Lentiviral Infections

All cell lines used in this study were purchased from the ATCC or European Collection of Authenticated Cell Cultures (A2780 cells). Cell lines under the passage number of 30 were used. All cell lines were tested negative for *Mycoplasma* during routine surveillance (IDEXX Bioanalytics) and reauthentication was not performed. MCF7 (female) cells were cultured in DMEM. HCC1569 (female), A2780 (female), and OVCAR-3 (female) cell lines were cultured in RPMI1640 medium. ES-2 (female) cells were cultured in McCoy's 5A medium. OV-7 (female) cells were cultured in a 1:1 mixture of DMEM/F12K medium. All media above contained 10% FBS, 2 mmol/L l-glutamine, 100 mg/mL penicillin, and 100 mg/mL streptomycin. UWB1.289 (female) and UWB1-BRCA1 (female) cells were cultured in a 1:1 mixture of RPMI1640 + MEGM containing 3% FBS, and UWB1-BRCA1 cells additionally were grown in 0.2 mg/mL G140 except when not used for experiments. Olaparib-resistant UWB1.289 cells were generated through long-term continuous treatment of parental UWB1.289 cells with successively increasing doses of olaparib over the course of several months until the 10-day olaparib IC_50_ of the pool of cells reached greater than 1 µmol/L. Following that, cells were routinely cultured in olaparib when not used for experiments. Lentiviral short hairpin RNA (shRNA) particles (Sigma-Aldrich) for PRMT5 and empty vector shRNA controls were purchased from Sigma. Lentiviral infections were performed through reverse infection by adding viral particles along with polybrene at 8 µg/mL during cell seeding and incubating for 24 hours. Cells were washed 3x with PBS and fresh medium containing 3 µg/mL Puromycin was added to wells. Cells were then incubated for an additional 4 days in the presence of puromycin before collecting cells for RNA extraction. Commercial drugs were used at indicated concentrations and included olaparib (Selleckchem; S1060), cisplatin (Selleckchem; S1166), 5-fluorouracil (5-FU; Selleckchem; S1209), and MS4322 (MedChem Express; HY-141877). Olaparib, 5-FU, and MS4322 were solubilized in DMSO. Cisplatin was solubilized in a sterile saline solution. C220 and PRT543 were kept in aliquots of 10 mmol/L stocks prepared in DMSO.

### RNA Sequencing and Bioinformatic Analyses

Cell pellets were washed in ice-cold PBS and stored at −80°C. RNA extraction, quantitation (NanoDrop and TapeStation), and RNA sequencing (RNA-seq; paired-end 2 × 150 bp sequencing at 100 million reads per sample Illumina) was performed by Azenta Inc. Differentially expressed gene (DEG) set filtered by *q* < 0.01 was used for gene set enrichment analysis (GSEA). Kyoto Encyclopedia of Genes and Genome (KEGG) pathway enrichment analysis was performed in WebGestalt (Lio and colleagues 2019, http://www.webgestalt.org/). Alternative splicing were analyzed in SUPPA (https://github.com/comprna/SUPPA).

### ChIP-qPCR

Chromatin immunoprecipitation (ChIP) assays were performed using the iDeal ChIP sequencing kit for Transcription Factors (Diagenode, catalog no. C01010055) according to the manufacturer's instructions. MCF-7 cells were treated for 4 days with C220. Following this incubation period, each treatment group (1.5 × 10^7^ cells) were fixed for 10 minutes at room temperature with 1% formaldehyde-containing medium. Nuclei were isolated, and the chromatin was sonicated with the Bioruptor Pico sonication device (Diagenode) for seven cycles (30 seconds ON, 30 seconds OFF). Sonicated chromatin was immunoprecipitated by incubation with anti-PRMT5 (07-405, Sigma-Aldrich/Millipore Sigma), anti-pICln (CLNS1A; ab192907, Abcam), anti-H4R3me2s (ab5823, Abcam), and IgG (3900 Cell Signaling Technology) antibodies overnight at 4°C. One percent of the chromatin used for each ChIP reaction was kept as input DNA. Immunoprecipitants were washed three times and eluted after treatment of proteinase K. Immunoprecipitated chromatin was subjected to qRT-PCR using SYBR Green master mix reagent (according to manufacturer's protocol) on a 384-well CFX qPCR (Bio-Rad). The percent input of enrichment was defined by the following formula: 2^[(Ctinput − 6.64) − Ctsample] * 100%.

### *In Vitro* Proliferation and Drug Combination Studies

Cell proliferation assays were performed by seeding cells at optimized cell densities in white sided 96-well plates (Greiner). Cells were allowed to attach overnight before dosing with compounds using a Tecan D300E liquid dispenser from diluted stock concentrations appropriate to maintain total DMSO in wells to less than 0.3%. All wells were normalized to the same percentage of DMSO. Assay plates were incubated at 37°C in an active humidified incubator with 97% humidity and 5% CO_2_. After 5 days, media was exchanged and fresh compound added. At day 10, plates were equilibrated to room temperature and media was completely removed and a 1:1 mixture of room temperature CellTiter-Glo reagent and medium was added to wells followed by shaking on an orbital shaker for 30 seconds. Plates were then incubated for 10 minutes, and luminescence was read on an Envision (PerkinElmer). To calculate cell viability, relative light unit (RLU) values for all wells were normalized to the average of multiple DMSO wells. SynergyFinder 3.0 was used to calculate ZIP synergy scores based on the average of three biological replicates.

### Western Blotting and qRT-PCR

Protein was extracted from cell pellets suspended in 1x PBS at a ratio of 1:3 volumes with 4% SDS lysis buffer containing 1X protease and phosphatase inhibitors, and 0.5% phenylmethylsulfonylfluoride (200 mmol/L, #8553S, Cell Signaling Technology). Lysates were transferred to Omega Biotek homogenizer columns (HCR003) and centrifuged at 10,000 rpm for 1.5 minutes. Protein was quantified with a Pierce bicinchoninic acid assay (Thermo Fisher Scientific, #23223, #23224) and 1X samples were prepared from 4X Laemmli sample buffer (Bio-Rad, #1610747), boiled for 5 minutes at 95°C, and run on 4%–15% SDS polyacrylamide gels (Bio-Rad). Semidry protein transfer to low fluorescence polyvinylidene difluoride membranes were done with a semidry Bio-Rad Trans-blot Turbo transfer system. Western blots were probed with antibodies via an iBind Flex Western device (Thermo Fisher Scientific) at manufacturer recommended concentrations. Fluorescent-tagged secondary antibodies were used for fluorescent detection (IR Dye 800CW Goat anti-rabbit, #926-32211; IR Dye 680RD Goat anti-mouse #926-68070; LI-COR) and were scanned on an Odyssey LI-COR CLx following a brief wash with water. For difficult to detect proteins, membranes were instead blocked in Odyssey LI-COR Intercept TBS blocking buffer (LI-COR, #927-60001) for 1 hour, washed in 1X TBST buffer (20X, 1% Tween-20, pH 7.4, Boston Bioproducts #IBB-181X) 3x for 5 minutes, probed overnight at 4°C in primary antibody solutions made in LI-COR Intercept TBS Antibody Diluent with tween 20 (LI-COR, #927-65001), incubated with secondary antibodies at a 1:20,000 dilution in Odyssey Licor TBS blocking buffer containing 0.01% SDS for 1 hour at room temperature, and washed 3x for 15 minutes in 1X TBST. Bands were quantified with Image Studio Ver 5.2. and normalized to appropriate loading controls.

RNA was extracted from cell pellets using Quick-RNA MiniPrep Plus Kit from Zymo Research (#R1058) as per manufacturer instructions. Eluted RNA was quantified by Nanodrop 8000 (ND8000P21H2) and normalized amounts of RNA were converted to cDNA by RT-PCR using Quanta Bio cDNA synthesis kit (#95047) and a Bio-Rad Thermal cycler (CFX connect Real-Time system) under the following conditions: 22°C/5 minutes, 42°C/30 minutes, 85°C/5 minutes. qRT-PCR was performed in triplicate using PerfecTa SYBR Green Supermix (QuantaBio, #95054-02K) in 384-well qPCR microplates (Applied Biosystems, #4483319) with the following program: 95°C/3 minutes, [39 cycles, 95°C/10 seconds, 60°C/30 seconds], 95°C/5 seconds. Quantitation was performed using the ddCT method with appropriate housekeeping genes.

### Immunofluorescence and HR Reporter Assay

Cells were seeded into chamber slides (Nunc Lab-Tek II, Thermo Fisher Scientific, #154534) and allowed to attach overnight before treating with compounds as indicated. Cells were fixed in 4% paraformaldehyde in PBS for 10 minutes at room temperature, followed by washes in ice-cold PBS. Permeabilization was done by incubating in 0.1% Triton X-100 in PBS for 10 minutes at room temperature, followed by washes in PBS. Cells were blocked in 1% BSA in PBS containing 0.1% Tween-20 at room temperature for 1 hour, and then washed in PBS. Next, cells were incubated for 1 hour at room temperature in primary antibody (1:200) against γH2AX (pS139)-Alexa Fluor 488 (Abcam, ab195188) in the dark. Nuclear and F-actin counterstaining were done by incubating with DAPI at 0.1 µg/mL for 10 minutes, and Texas Red-X Phalloidin (Thermo Fisher Scientific, T7471) at the manufacturer recommended dilution for 30 minutes, respectively. Imaging was conducted on a Revolve R4 (Echo). ImageJ 1.54f was used for image preparation and quantitation. Total γH2AX foci/field were quantitated by the Find Maxima feature. Average foci/nucleus represents total maxima/total nuclei.

For the HR reporter assay, HeLa cells stably expressing HR-GFP were purchased from TopoGen. Cells were treated with C220 for 4 days, followed by transfection with I-Sce1 plasmid. Cells were fixed, permeabilized, and counterstained with DAPI and phalloidin as written above before imaging and counting the percentage of GFP-positive cells from total.

### Xenograft Models

Cell line–derived xenograft (CDX) studies in A2780 ovarian cancer xenografts were conducted at Crown Bioscience. The A2780 cell line mixed 1:1 with Matrigel was inoculated subcutaneously into 6 to 9 weeks old female BALB/c nude mice (GemPharmatech Co., Ltd). For drug treatment studies, tumors were grown in engrafted mice until reaching 80 to 120 mm^3^ in size. Mice were then randomized into treatment groups (*n* = 8 mice/group) to administer PRT543 (15 or 40 mg/kg chow daily) or olaparib (100 mg/kg oral gavage daily). Patient-derived xenograft (PDX) studies were conducted at Champions Oncology. For all PDX models, stock mice were bilaterally implanted with fragments from Champions TumorGraft models representing human ovarian (CTG-0703, CTG-1086) and human breast (CTG-0869, CTG-1242) cancer (Champions Oncology). RNA-seq and whole-exome sequencing data for the PDX models are available from Champions Oncology's database (https://lumin.championsoncology.com). Upon reaching 1,000 to 1,500 mm^3^ in size, tumors were harvested and implanted subcutaneously in the left flank of 6 to 8 weeks old female athymic Nude-Foxn1nu (immune-compromised) mice (Envigo). For drug treatment studies, tumors were grown in engrafted mice until reaching 150 to 300 mm^3^ in size. Mice were then matched by tumor size and assigned into control or treatment groups (*n* = 10 mice/group) to administer PRT543 (40 mg/kg chow 5 days on/2 days off) or olaparib (50 mg/kg oral gavage daily). All experiments and procedures were approved by the Institutional Animal Care and Use Committee of Crown Biosciences or Champions Oncology.

### Data Availability

The data generated in this study may be available upon request from the corresponding author. Raw sequence data were deposited in a repository in Sequence Read Archive (SUB13835362; SUB13835665; SUB13830138).

## Results

### PRMT5 Gene Expression Positively Correlates to DDR-related Gene Expression in Cancer Cells

**FIGURE 1:**
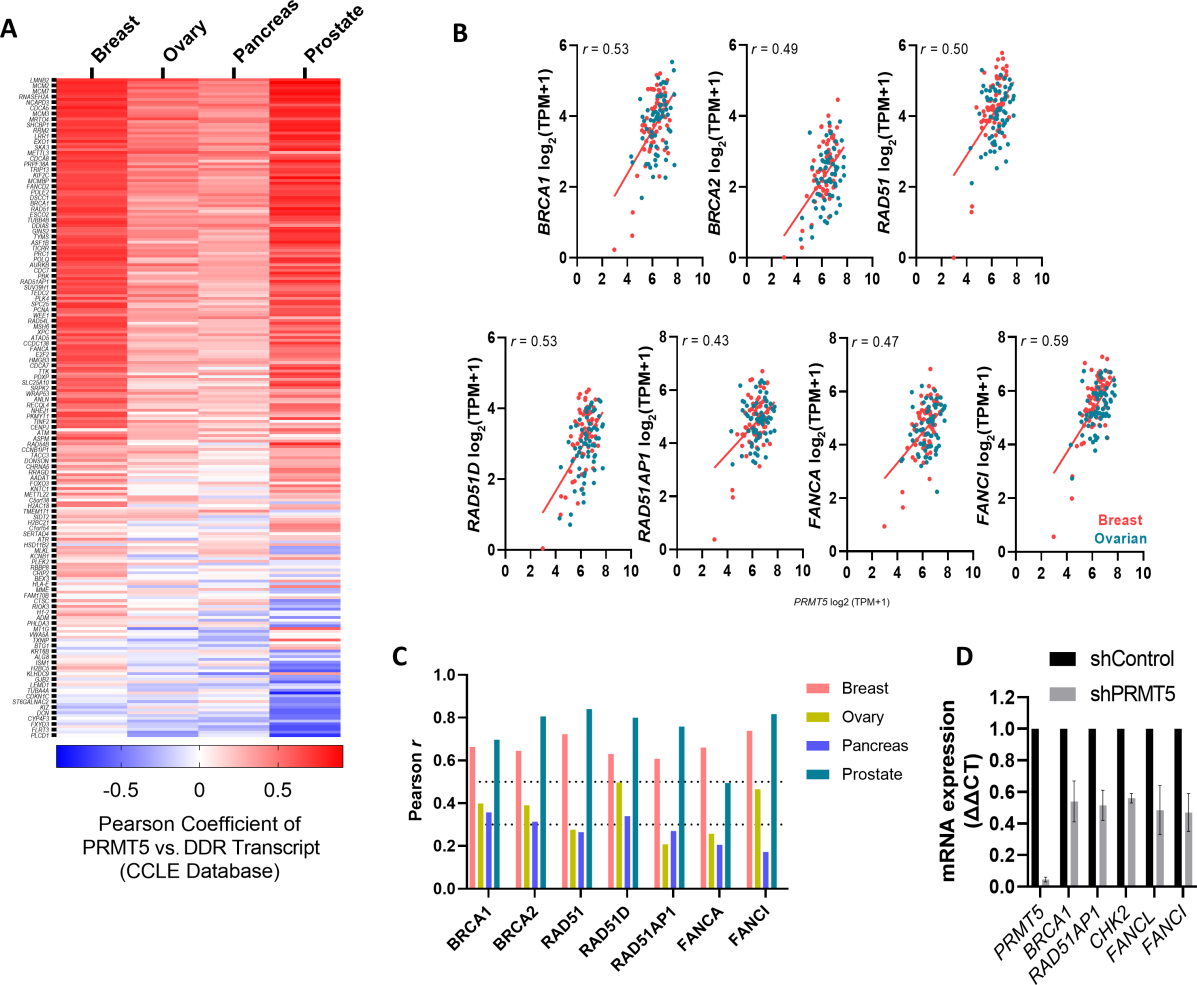
PRMT5 gene expression positively correlates to DDR gene expression in cancer cells. **A,** Heat map of Pearson correlation coefficients between PRMT5 and HRD signature genes for breast, ovarian, pancreatic, and prostate cancer cell lines from CCLE (heat map is sum ranked). **B,** Dot plots showing PRMT5 expression relative to key DDR genes in ovarian and breast cancer cell lines (dots represent cell lines). **C,** Pearson correlation coefficient *r* values were deconvoluted and shown in bar graph. **D,** mRNA expression of select correlated DDR genes by qPCR following transient genetic knockdown of PRMT5 by shRNA in MCF7 cells (*n* = 2).

To broadly evaluate a connection between PRMT5 and DDR pathway regulation in clinically relevant tumor types, we first evaluated the correlation of *PRMT5* mRNA expression with that of 235 genes associated with DNA replication and repair across 188 cancer cell lines (61 breast, 64 ovarian, 11 prostate, and 52 pancreatic cancer cell lines) from the Cancer Cell Line Encyclopedia (CCLE) database (33). The expression of *PRMT5* broadly and frequently positively correlated with that of a wide range of DNA repair–related genes (Fig. 1A). *PRMT5* expression was moderately to strongly correlated (Pearson score ≥ 0.4) with established DDR genes such as *BRCA1*, *BRCA2*, *RAD51*, *RAD51D*, *RAD51AP1*, *FANCA*, and *FANCI* across 125 ovarian and breast cancer cell lines (Fig. 1B). Among the four types of cancers analyzed, breast and prostate cancer cell lines show a stronger correlation between PRMT5 and DDR gene expression (Fig. 1C). In MCF7 breast cancer cells, we validated transcript reductions in select correlated genes by qRT-PCR following transient knockdown of PRMT5 by shRNA (Fig. 1D), demonstrating a functional significance of the positive correlation.

### C220 Broadly Suppresses DDR Gene Expression in Ovarian and Breast Cancer Cells

**FIGURE 2:**
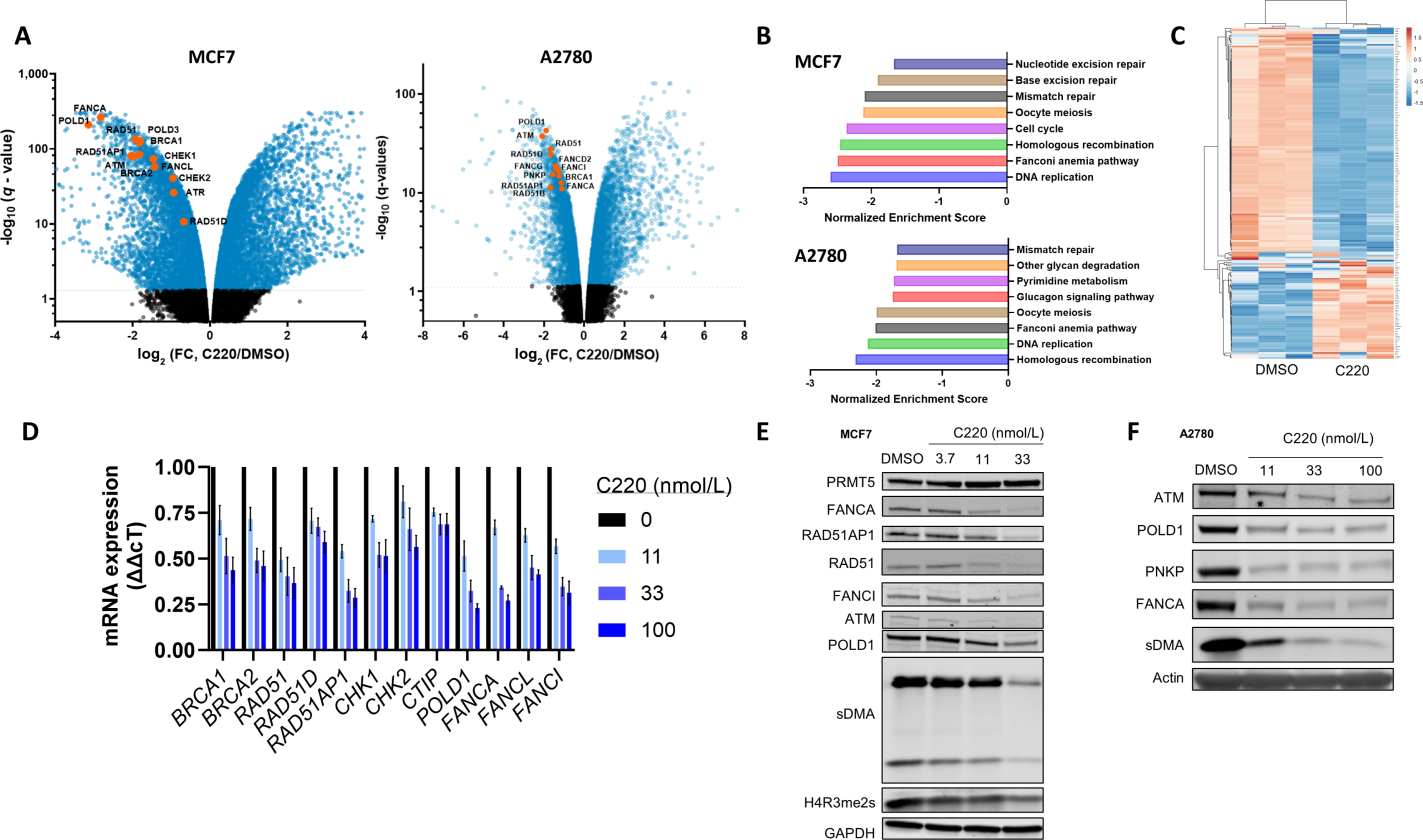
C220 broadly suppresses DDR Gene expression in ovarian and breast cancer cells. **A,** Volcano plots from RNA-seq data in breast (MCF7) and ovarian (A2780) cancer cell lines treated with 100 nmol/L C220 for 4 days (*n* = 3). Select DDR genes are highlighted. **B,** Top eight downregulated hits from KEGG pathway analysis of significantly DEGs from MCF7 and A2780 RNA-seq data (*q* value < 0.01, *n* = 3). **C,** Clustered heat map of HRD signature genes in C220-treated MCF7 cells (*n* = 3). **D,** Dose-dependent downregulation of select DDR genes was validated by qRT-PCR (fold change ddCT from DMSO control) following treatment with C220 for 4 days (*n* = 3). Data are represented as mean ± SEM. **E** and **F**, Western blots highlighting dose-dependent downregulation of select DDR proteins following treatment with C220 for 4 days (*n* = 2).

Previously, we reported that C220 is a potent and highly selective small-molecule inhibitor of PRMT5 (ref. 16; Supplementary Fig. S1A–S1C). To evaluate the molecular profile of C220, we evaluated changes in global gene expression by RNA-seq following C220 treatment in HR-proficient breast (MCF7) and ovarian (A2780), and HR-deficient breast (HCC1569; *BRCA2 mut*) cancer cell lines. Treatment with C220 robustly altered the global gene expression profile in all cell lines tested (Fig. 2A; Supplementary Fig. S2A). Among the significantly downregulated genes were established DDR genes such as *BRCA1, RAD51, RAD51D, RAD51AP1, ATM, ATR, FANCA, FANCL, POLD1*, and *PNKP*. Furthermore, a GSEA revealed significant downregulation of DNA replication and repair, FA, and HR pathways in HR-proficient MCF7 and A2780 cell lines treated with C220 (Fig. 2B). In *BRCA2*-mutated HCC1569 cells, C220 moderately regulated FA and HR pathways (Supplementary Fig. S2B). We next performed a cluster analysis using an HRD gene signature established by (34), which revealed a profound impact of C220 on the expression of HR-associated genes in MCF7 cells (Fig. 2C). To validate several of these changes, we treated MCF7 and A2780 cells with C220, which revealed a dose-dependent decrease in mRNA expression of >10 DDR-related genes (Fig. 2D). Notably, a concomitant decrease in the expression of corresponding proteins was validated for several of these genes in both breast and ovarian cancer cells (Fig. 2E and F). Treatment with C220 resulted in potent downregulation of sDMA, a canonical pharmacodynamic marker for PRMT5 inhibition. Interestingly, C220 partially reduced the expression of global H4R3me2s (Fig. 2E), which has been reported as one mechanism of a PRMT5-mediated DNA damage response in prostate cancer (14). C220 also broadly decreased DDR gene expression in other cancer types such as uveal melanoma and head and neck cancer (Supplementary Fig. S2C). In an I-Scel reporter assay, C220 treatment shows a trend of reducing HR efficiency in cancer cells (Supplementary Fig. S2D and S2E). Our data support a conserved role for PRMT5 in regulating expression of DDR-realted and DNA replication–related genes in cancer cells, which can be blunted by PRMT5 inhibitors.

### C220 Dysregulates Chromatin- and Splicing-mediated Regulation of DDR Pathways

**FIGURE 3:**
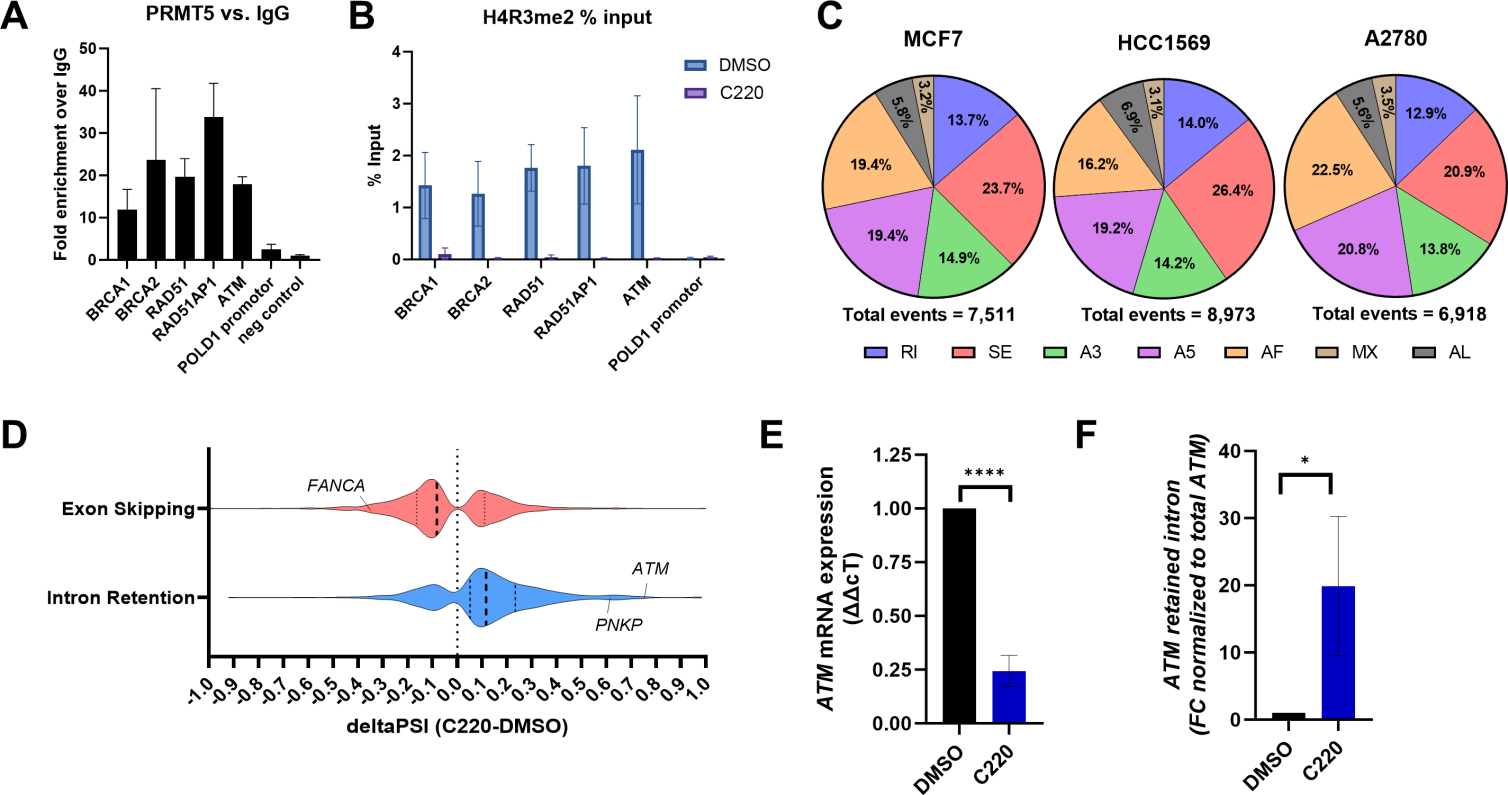
C220 dysregulates chromatin- and splicing-mediated regulation of DDR pathways. **A,** ChIP-qPCR assays using primers against the proximal promoter of indicated DDR genes following capture of chromatin-bound PRMT5 in nontreated MCF7 cells. Data are shown as fold enrichment over IgG (*n* = 3). **B,** ChIP-qPCR assays using primers against the proximal promoter of DDR genes following capture of chromatin-bound H4R3me2 from MCF7 cells treated with 250 nmol/L C220 for 4 days. Data are shown as % input (*n* = 3) **C**, AS analysis of RNA-seq data from MCF7, HCC1569, and A2780 cells following treatment with 100 nmol/L C220 for 4 days. Total events represent the total number of AS with statistical significance (*P* < 0.05). RI = retained intron, SE = skipped exon, A3 = alternative 3′ splice-site, A5 = alternative 5′ splice site, MX = mutually exclusive exons, AF = alternative first exon, AL = alternative last exon. **D,** Violin plot of global intron retention and exon skipping events induced by C220 in A2780 cells annotated with select DDR genes with significant (*P* < 0.05) events. **E** and **F**, qPCR of total *ATM* or *ATM* retained intron in A2780 cells treated with 100 nmol/L C220 for 4 days.

Previous studies reported PRMT5 epigenetic regulation of either histones or core spliceosome factors as distinct potential mechanisms associated with PRMT5 regulation of DNA repair pathways (14, 20). Thus, we sought to contextualize mechanistically how C220 may inhibit DDR gene expression. The partial reduction in global expression of H4R3me2s by C220 suggested PRMT5 could be regulating gene expression by altering chromatin accessibility through this axis. To test this, we performed ChIP of PRMT5 followed by qPCR to assess the presence of PRMT5 at the promoter regions of several DDR genes in MCF7 cells. We found PRMT5 was enriched at the proximal promoter regions of the DDR genes *BRCA1*, *BRCA2*, *RAD51*, *RAD51AP1,* and *ATM* (Fig. 3A). To confirm enrichment of PRMT5 at these genes, we repeated the ChIP assay comparing wildtype MCF7 cells and PRMT5 partially depleted MCF7 cells using the PRMT5 degrader MS4322 (35). Depletion of PRMT5 subsequently resulted in a reduction in PRMT5 fold change enrichment over IgG at the proximal promotor regions of DDR genes (Supplementary Fig. S3A and S3B). Similarly, we verified the enrichment of H4R3me2s at these same promoters by ChIP-qPCR, which was blocked by treatment with C220 (Fig. 3B). These data suggest basal regulation of *BRCA1*, *BRCA2*, *RAD51*, *RAD51AP1,* and *ATM* gene expression by PRMT5 functions at least in part through PRMT5-catalyzed H4R3me2s at proximal promoters of these genes. Interestingly, the PRMT5 cofactor chloride nucleotide-sensitive channel 1A (pICLn) is reported to play a role in PRMT5-H4R3me2–mediated gene regulation in prostate cancer (14). To gain insight into this, we tested whether pICLn was present at the *BRCA1* promoter by ChIP-qPCR. Indeed, pICLn was found at the *BRCA1* promoter in control cells, and this was lost by treatment with C220 (Supplementary Fig. S3C), generally supporting previous reports suggesting PRMT5 plays a role in recruitment of pICLn to DDR gene promoters (14).

PRMT5 has been closely linked to AS and various mechanistic connections to DDR pathways have been implicated previously. For example, PRMT5 regulation of *POLD1* and *ATM* via AS have been previously suggested by us and others in leukemias and uveal melanomas (18, 36). To broadly assess whether splicing dysregulation was a molecular feature of C220-treated cells, we analyzed AS changes within our RNA-seq datasets in ovarian and breast cancer cell lines. Indeed, C220 induced assorted global AS events (MCF7: 7511, HCC1569: 8973, A2780: 6918 significant events; Fig. 3C). The most frequent associated events across all cell lines were exon skipping (≥21%), alternative 5′ splice sites (>19%), alternative first exons (>16%), alternative 3′ splice sites (>13%), and retained introns (≥13%; Fig. 3C). Exon loss and intron retention are associated with disrupted gene (e.g., by nonsense-mediated decay) and protein expression (37). Global analysis of genes with exon skipping or intron retention events following treatment of A2780 cells with C220 is represented by the violin plot in Fig. 3D. Among significant exon skipping events (FDR < 0.05; dPSI < −0.1), we identified *FANCA* (Fig. 3D), for which we have shown a substantial reduction in protein expression induced by C220 (Fig. 2E and F). In addition, among significant retained intron events (FDR < 0.05; dPSI < −0.1), we identified *ATM* and *PNKP* (Fig. 3D), which also are strongly downregulated by C220 (Fig. 2E and F). The induction of *ATM* retained intron between exon 32 and 33 was detected by qRT-PCR (Fig. 3E and F). Interestingly, *POLD1* intron retention did not reach significance, although previous works have linked AS of this to serine and arginine rich splicing factor 1 (SRSF1; ref. 18), and preliminary reports highlight PRMT5 inhibition induced *POLD1* intron retention in other datasets (uveal melanoma; refs. 18, 36). Notably, we observed relatively less PRMT5 and H4R3me2s at the *POLD1* proximal promoter, despite marked gene and protein downregulation by C220, suggesting PRMT5 may indeed regulate *POLD1* expression via AS (Fig. 3A and B). Collectively, our data suggest PRMT5 plays a dual role in regulating DDR in a gene-specific context both by epigenetically controlling gene expression through histone methylation at DDR gene promoters, and through regulation of DDR mRNA splicing. Importantly, pharmacologic inhibition of PRMT5 functionally downregulates key DDR genes regulated through either mechanism, demonstrating the broad impact of C220 on DDR and replication pathways.

### PRMT5 Inhibition Induces DNA Repair Deficiency, and Synergizes with PARP Inhibition and Chemotherapy *In Vitro* and *In Vivo*

**FIGURE 4:**
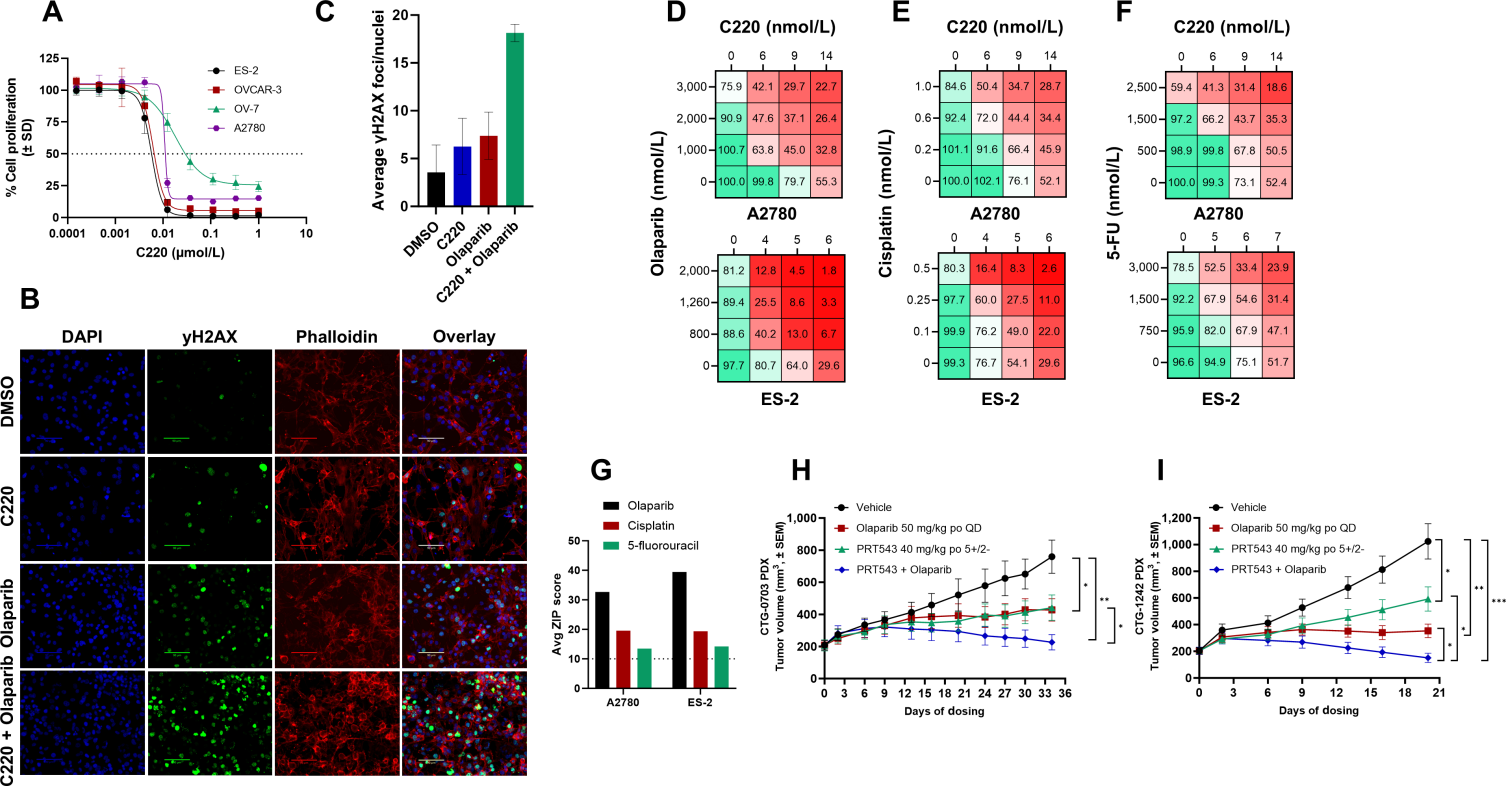
PRMT5 inhibition induces DNA repair deficiency, and synergizes with PARP inhibition and chemotherapy *in vitro* and *in vivo*. **A,** Cell proliferation of HR-proficient ovarian cancer cell lines was assessed by cell-titer glo assays following 10 days treatment with C220 (*n* = 3). **B,** Immunofluorescent detection of γH2AX foci in ES-2 cells treated with C220, olaparib, or in combination for 4 days. Images are taken at 20x and are representative of two biological replicates. **C,** Quantitation of average γH2AX foci/nuclei (>250 nuclei/condition, *n* = 2). **D–F,** Cell proliferation assays in ovarian cancer cell lines following treatment with olaparib, cisplatin, 5-FU, or C220 alone or in combination for 10 days. Data represent percent cell proliferation relative to DMSO controls and the average response of three biological replicates is shown. **G,** ZIP scores were calculated from average percent cell proliferation data from drug combination using SynergyFinder. **H,***In vivo* mouse ovarian cancer PDX (CTG-0703, *BRCA1* deletion) treated with PRT543, olaparib, or in combination (*n* = 10 mice/group). **I,***In vivo* mouse breast cancer PDX (CTG-1242, HR proficient) treated with PRT543, olaparib, or in combination (*n* = 10 mice/group). Data are represented as mean ± SEM. H and I, asterisks indicate statistical significance noted by connecting bars (*, *P* <0.05; **, *P* <0.01) using a Mann–Whitney test.

Given that PRMT5 inhibition downregulates expression of genes associated to DDR and DNA replication, we postulated that combining PRMT5 inhibitors (C220 or PRT543) with agents targeting DNA damage, repair, and replication mechanisms might be a viable therapeutic strategy in tumors regardless of genomic alterations in DDR genes. First, we evaluated the proliferation of several HR-proficient ovarian cancer cell lines following treatment with C220 alone for 10 days. Baseline sensitivity to C220 was strikingly high for all cell lines, with cellular IC_50_ values between 3 and 18 nmol/L (Fig. 4A). To understand the potential functional consequence of combining C220 with such agents, we evaluated the induction of DNA damage when combined with the PARP inhibitor, olaparib. In ES-2 cells, the combination of C220 with olaparib increased nuclear γH2AX foci formation (Fig. 4B and C), suggesting an increase in DNA damage consequent to DNA repair deficiency. We then evaluated the phenotypic impact by performing drug combination studies with mechanistically relevant and clinically approved molecules in HR-proficient ovarian cancer cell lines. We found the combination of C220 with either olaparib (Fig. 4D; Supplementary Fig. S4A), cisplatin (Fig. 4E; Supplementary Fig. S4B), or 5-FU (Fig. 4F; Supplementary Fig. S4C) led to a robust and synergistic (Fig. 4G; Supplementary Fig. S4D) antiproliferative effect in multiple cell lines (A2780, ES2, OV7). These data suggested potential utility in combining PRMT5 inhibitors with anticancer drugs targeting DNA damage, repair, and replication mechanisms.

Next, we sought to translate these *in vitro* findings to clinically relevant PDXs. To do so, we tested the combination of PRT543 with olaparib in an ovarian cancer PDX model (CTG-0703, *BRCA1* mutated). PRT543 is an orally bioavailable potent and selective PRMT5 inhibitor, which is active in human and rodent models (37). PRT543 shows improved oral pharmacokinetics in rodents, compared with C220. The potency and selectivity of PRT543 analyzed in biochemical and cellular assays are comparable with that of C220 (Supplementary Fig. S1). Mice harboring xenografts were treated orally with either 50 mg/kg olaparib or 40 mg/kg PRT543, both of which induced significant tumor growth inhibition as single agents. Combination of PRT543 with olaparib resulted in a further significant reduction in tumor growth (24% tumor growth inhibition (TGI) improvement over olaparib monotherapy; Fig. 4H). As proof of concept, we then expanded our *in vivo* studies to test PRT543 in a breast cancer PDX model (CTG-1242, HR proficient). Once again, mice harboring xenografts were treated orally with either 50 mg/kg olaparib or 40 mg/kg PRT543. Both olaparib (TGI 78%) and PRT543 (53% TGI) therapy inhibited tumor growth as single agents (Fig. 4I). Once again, combination of PRT543 with olaparib resulted in a further significant reduction in tumor growth (28% TGI improvement above olaparib monotherapy; Fig. 4I). Consistent with the expected pharmacodynamic readout, PRT543 inhibited tumor sDMA as measured by Western blot analysis, in all groups where it was included (Supplementary Fig. S4E and S4F). Collectively, our data provide compelling evidence *in vitro* and *in vivo* that PRMT5 inhibitors could be used in combination clinically approved PARP inhibitors and chemotherapies for the treatment of ovarian or breast cancer, potentially irrespective of genomic HRD status.

### PRT543 Inhibits Growth of PARP Inhibitor–resistant Ovarian and Breast Cancer Models *In Vitro* and *In Vivo*

**FIGURE 5:**
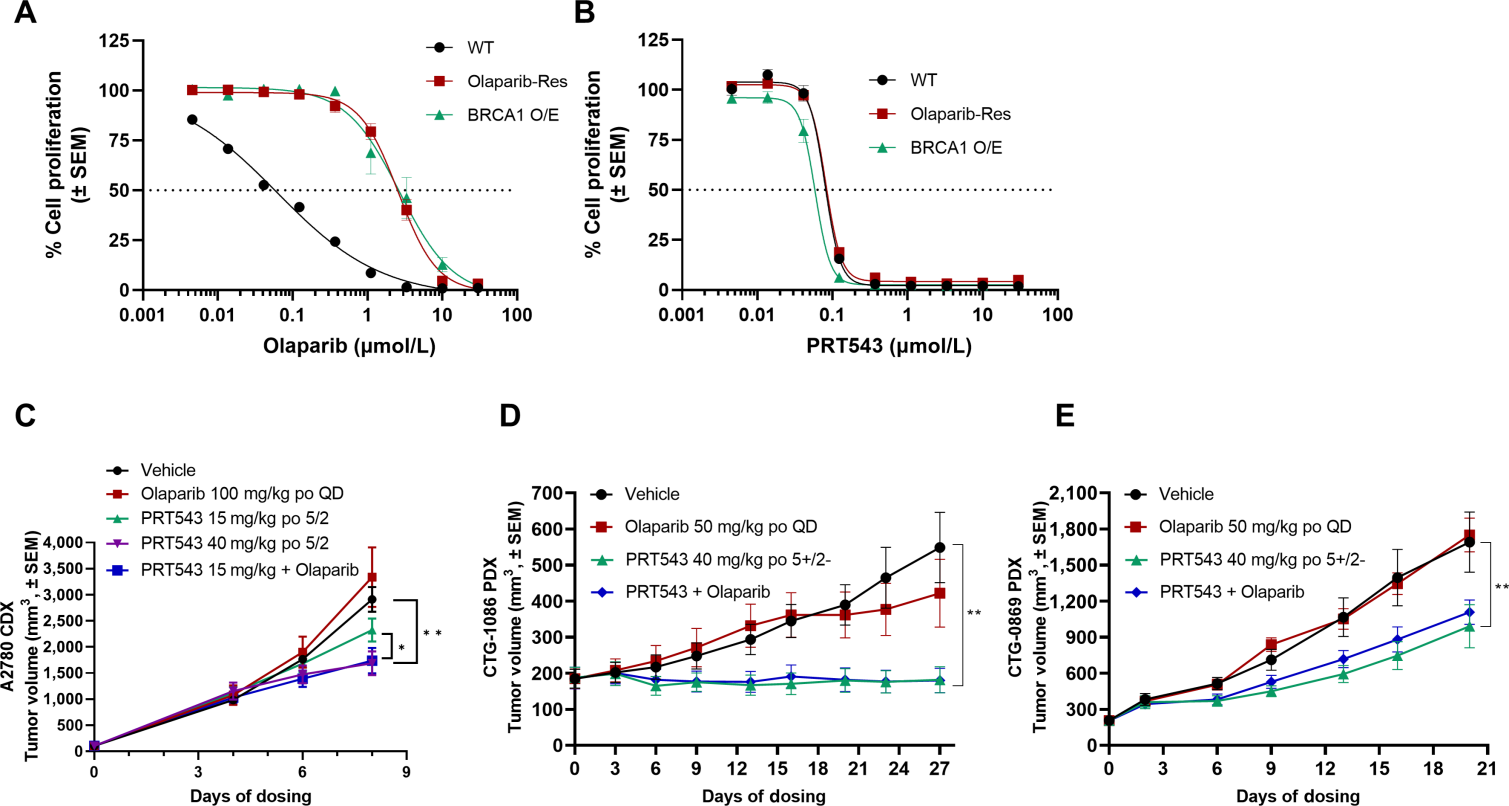
PRT543 inhibits growth of PARP inhibitor–resistant ovarian and breast cancer models *in vitro* and *in vivo*. Cell proliferation of UWB1.289 parental, BRCA1-overexpressing, and olaparib-resistant cell lines was assessed by cell-titer glo assays following 10 days treatment with olaparib (**A**) or PRT543 (**B**; *n* = 3). **C,***In vivo* mouse CDX of A2780 ovarian cancer cells treated with PRT543, olaparib, or in combination (*n* = 8 mice/group). **D,***In vivo* mouse ovarian cancer PDX (CTG-1086, *BRCA1* deletion) with acquired resistance to olaparib was treated with PRT543 or olaparib (*n* = 10 mice/group). **E,***In vivo* mouse breast cancer PDX (CTG-089, *BRCA1/2* hemizygous deletion) with acquired resistance to olaparib was treated with PRT543 or olaparib (*n* = 10 mice/group). A–E, Data are represented as mean ± SEM. C–E, Asterisks indicate statistical significance from vehicle (**, *P* <0.01) using a Dunnett test.

To address our hypothesis that broad impact on DDR gene regulation could lead to circumvention of PARP inhibitor resistance, we interrogated several internally and externally generated olaparib-resistant models, both *in vitro* and *in vivo*. The UWB1.289 ovarian cancer cell is HR deficient, harboring a *BRCA1* mutation. In contrast, an established variant of this cell line made to re-express BRCA1 exogenously has been characterized previously (38). We first verified that the UWB1.289-BRCA1 cell line had increased resistance to olaparib in a 10-day cell proliferation assay, which showed a 43-fold reduction in cellular IC_50_ (2.7 µmol/L) compared with parental cells (Fig. 5A). We then tested the sensitivity of this paired model to PRT543 in the same assay. As expected, parental UWB1.289 cells were highly sensitive to PRT543 (IC_50_ < 80 nmol/L). Strikingly, UWB1.289 BRCA1-expressing cells retained complete sensitivity to PRT543 compared with parental cells (Fig. 5B), suggesting PRT543 can circumvent resistance mechanisms associated with the re-expression of BRCA1. To further interrogate this, we next generated UWB1.289 cells with acquired olaparib resistance by continuous treatment of parental cells to increasing doses of olaparib until we obtained a pool of cells that had > 1 µmol/L resistance to olaparib (“UWB1.289 Olaparib-Res”). This cell line exhibited comparable resistance to olaparib as BRCA1-overexpressing cells, with an IC_50_ of 2.6 µmol/L (Fig. 5A). Remarkably, UWB1.289 Olaparib-Res cells had equipotent sensitivity to PRT543 compared with parental cells (Fig. 5B). Furthermore, pretreatment of olaparib-resistant cells with PRT543 for 4 days, followed by 6 days cotreatment with a sub-IC_50_ dose of PRT543 with 1 µmol/L olaparib completely blunted cell proliferation (Supplementary Fig. S5A). Therefore, our *in vitro* data suggest treatment with PRT543 can overcome olaparib resistance associated with drug exposure or re-expression of genetically lost DDR genes.

We next sought to understand better the potential clinical translatability of these *in vitro* findings. To test this, we evaluated the *in vivo* efficacy of PRT543 in olaparib-resistant ovarian CDX and PDX models. First, mice were inoculated with A2780 ovarian cancer cells (*BRCA1/2* WT) followed by oral treatment with olaparib, PRT543, or in combination. Treatment of mice with 100 mg/kg olaparib had no appreciable impact on tumor growth, whereas treatment with PRT543 (40 mg/kg) significantly inhibited tumor growth (56.2% TGI; Fig. 5C). Combination of olaparib with a lower dose of PRT543 (15 mg/kg) resulted in significant reduction in tumor growth (24.6% TGI improvement over monotherapy) compared with PRT543 alone at an equivalent dose (Fig. 5C). Next, we evaluated the activity of PRT543 in an ovarian cancer PDX model which had acquired resistance to olaparib (CTG-1086, *BRCA1* deleted). Treatment of mice harboring this xenograft with 50 mg/kg of olaparib for over 3 weeks resulted in no significant inhibition in tumor growth (Fig. 5D). Conversely, treatment of mice with 40 mg/kg PRT543 as a single agent led to robust tumor growth inhibition versus control mice (Fig. 5D; TGI = 102%), and reduced tumor sDMA (Supplementary Fig. S5B and S5C). Finally, we again sought to evaluate the translation of these findings to models of olaparib resistance in other clinically relevant tumors, and thus tested the sensitivity of an olaparib-resistant breast cancer PDX (CTG-0869, *BRCA1/2* hemizygously deleted) to PRT543. Treatment of mice for three weeks with olaparib had no effect on tumor growth. Consistent with our *in vitro* and *in vivo* findings in ovarian cancer models, treatment with PRT543 significantly reduced tumor growth (TGI = 52%) as a single agent (Fig. 5E) and reduced tumor sDMA (Supplementary Fig. S5B and S5C). Thus, our data highlight for the first time the potential utility of clinically relevant PRMT5 inhibitors for the treatment of PARP inhibitor–resistant ovarian and breast cancers.

In conclusion, our data provide mechanistic insight into clinically relevant PRMT5 inhibitors, which potently, broadly, and functionally suppress DDR pathways. Our work suggests PRMT5 inhibitors can target DDR genes regulated through histone-mediated chromatin interactions or through induction of detrimental AS. In this context, our work highlights a new layer of therapeutic potential for PRMT5 inhibitors, which may have expanded utility in ovarian and breast cancers without mutations in HR genes when combined with PARP inhibitors or chemotherapy, as well as for the treatment of patients with tumors that have developed resistance to PARP inhibitors.

## Discussion

The approval of clinical drugs that take advantage of synthetic lethal vulnerabilities have underscored the preclinical and clinical value of precision oncology. Tactically selecting patients likely to respond to therapies based on genomics, as is the case for PARP inhibitor treatment in *BRCA1/2*-deficient tumors, offers an opportunity to maximize drug efficacy while limiting toxicity. Similarly beneficial is the identification of rational drug combination strategies that chemically mimic synthetic lethality. Identifying new routes to utilize precision therapeutics remains an important unmet clinical need. In this work, we highlighted evidence that PRMT5 inhibitors broadly and potently downregulate core DDR pathways in ovarian, breast, and other cancers resulting in a DNA repair–deficient phenotype. In this context, we describe the *in vitro* and *in vivo* utility of combining preclinical and clinical stage PRMT5 inhibitors with PARP inhibitors and standard-of-care chemotherapies for the treatment of ovarian and breast cancers.

PRMT5 dysregulation occurs in ovarian, breast, and other malignancies, correlating with poor outcome and disease progression (5–8). Although several PRMT5 inhibitors have entered clinical trials for solid and hematologic tumors (9–11), improving the therapeutic index of epigenetics-based targeted therapies is paramount due to the genome-wide impact of these regulators. *MTAP* deletions (39), splicing mutations (17, 40), and more recently genetically defined defects in HR genes (41) have been of significant interest to the development of PRMT5 inhibitors for these reasons. With respect to the latter, our work and that of several other groups point to a master regulatory role for PRMT5 in regulating DNA replication and repair. In prostate cancer, Owens and colleagues demonstrated a unique mechanism by which PRMT5 and cofactor pICln coordinate H4R3me2s-mediated transcriptional activation at DDR promoters in response to irradiation (14). Our data support PRMT5, in part, could regulate the expression of *BRCA1, BRCA2*, and *RAD51* through a similar axis, and suggest a significant role for PRMT5 in maintaining basal expression of these genes. Interestingly, global gene expression analyses following PRMT5 inhibition also revealed enriched downregulation of DDR pathways in concert with significant and preferential AS of DDR genes. This is consistent with the role of PRMT5 in the regulation of pre-mRNA splicing and the enrichment of DDR-related splicing in blood and brain cancers (19, 20, 31, 32). Consequently, intron retention and exon skipping events can downregulate transcript (NMD) and protein expression. We previously observed intron retention in *POLD1* (exon 22–23), *PNKP* (exon 19–20), *ATM*, and *ATR* in uveal melanoma cell lines treated with PRT543 with concomitant loss in protein expression (36). Intriguingly, PRMT5-mediated AS of *POLD1* and *PNKP* are associated with core spliceosome factor SRSF1 (18), whereas *ATR* splicing has been linked to splicing factor 3b subunit 1 (SF3B1) mutant cells (42). To our knowledge, we are the first to identify PRMT5-mediated AS events in *ATM*. Future studies warrant defining cross-talk mechanisms between direct chromatin regulation and spliceosome-mediated epigenetic regulation of DDR genes by PRMT5.

Clinically actionable opportunities associated with PRMT5 regulation of DDR genes have been largely unexplored, although several studies have indicated susceptibility of blood and prostate cancers to *ATM* inhibitors (16) or irradiation (14). PRMT5 is reported to positively correlate with DDR genes in 32 The Cancer Genome Atlas clinical datasets (14), which translates well to correlations we observed in *in vitro* cancer models (CCLE database; Fig. 1A–C). Here we demonstrate preclinical proof of concept that the PRMT5 inhibitor C220 synergizes with therapies targeting DNA repair and replication (PARP inhibition, cisplatin, 5-FU) in HR-proficient models. Several other studies support this hypothesis. For example, recent work by Du and colleagues (2021) highlights the benefit of combining PRMT5 ablation with DNA damage–inducing therapies such as interstrand crosslinks (ICL) agents in glioblastoma (30). PRMT5 ablation alone also recapitulates FA pathway deficiency seen in *MTAP-*deleted tumors (30), mimicked by the use of MAT2A inhibitors in *MTAP-*deleted cancers that was found to be synergistic with docetaxel in squamous lung and pancreatic PDX models (43). Finally, during the preparation of this article, Xie and colleagues (2023) reported reduced colony formation of A2780 cells when treated in combination with a single dose of PRMT5 inhibitor DW14761 with olaparib, cisplatin, or 5-FU (44). Importantly, our investigational clinical PRMT5 inhibitor PRT543 displayed significant *in vivo* efficacy when combined with olaparib in multiple preclinical CDX and PDX ovarian and breast cancer models.

Patients with advanced disease often develop resistance to targeted therapies, such as PARP inhibitors, which may occur through circumventing of DDR pathways or re-expression of genetically lost proteins. We postulated that the broad and potent suppression of DDR pathways may blunt PARP inhibitor resistance mechanisms by either inhibiting alternatively activated DDR signaling or via suppression of re-expressed DDR proteins. Strikingly, our data suggest PRT543 potently inhibits olaparib-resistant ovarian cancer cell line models with endogenous *BRCA1* mutation that have been engineered to re-express BRCA1, as well as long-term drug-induced acquired resistance to olaparib. Moreover, this translated to patient-derived ovarian and breast cancer *in vivo* models of acquired olaparib resistance, highlighting a potential utility of PRT543 in treating PARP inhibitor–resistant tumors. Further studies profiling molecular changes in these models will be important to help define the underlying mechanisms associated with overcoming PARP inhibitor resistance through PRMT5 inhibition. In conclusion, we have identified mechanisms associated with novel and clinically relevant PRMT5 inhibitors that disrupt a multifaceted DNA replication and repair regulatory program driven by PRMT5 and induce a DNA repair–deficient phenotype. Our work identifies new and potentially clinically actionable sensitivities in ovarian and breast cancers through combination of PRMT5 inhibitors with clinically approved drugs and as a single agent in PARP inhibitor–resistant cancers.

## Supplementary Material

Figure S1 Selectivity and potency of PRMT5 inhibitors analyzed by biochemical or cellular assays

Figure S2 C220 broadly suppresses DDR gene expression in cancer cells and reduces DNA repair.

Figure S3 ChIP-PCR validation and pICln ChIP-PCR

Figure S4 PRMT5 inhibitors synergize with PARP inhibition and chemotherapy in breast and ovarian cancer cells in vitro and in vivo.

Figure S5 PRT543 inhibits growth of PARP inhibitor resistant ovarian and breast cancer models in vitro and in vivo.
