# Supplementary figures and images for: PRMT5 Inhibitors Regulate DNA Damage Repair Pathways in Cancer Cells and Improve Response to PARP Inhibition and Chemotherapies
Source: Cancer Res Commun. 2023 Nov 6;3(11):2233–43. doi: 10.1158/2767-9764.CRC-23-0070 (PMC10627093; doi:10.1158/2767-9764.CRC-23-0070)

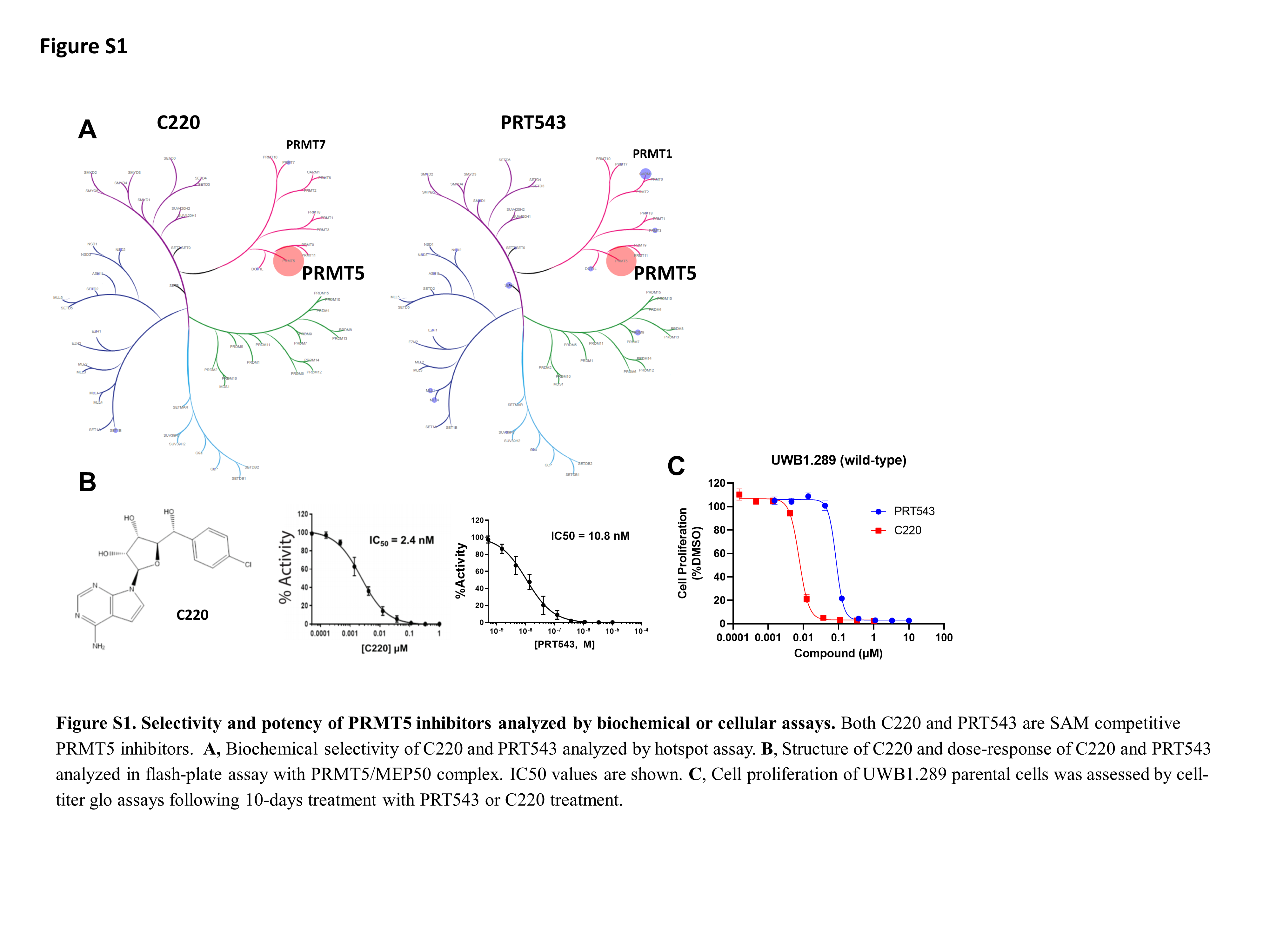

Supplement: Figure S1 — Selectivity and potency of PRMT5 inhibitors analyzed by biochemical or cellular assays [file crc-23-0070-s01.png]

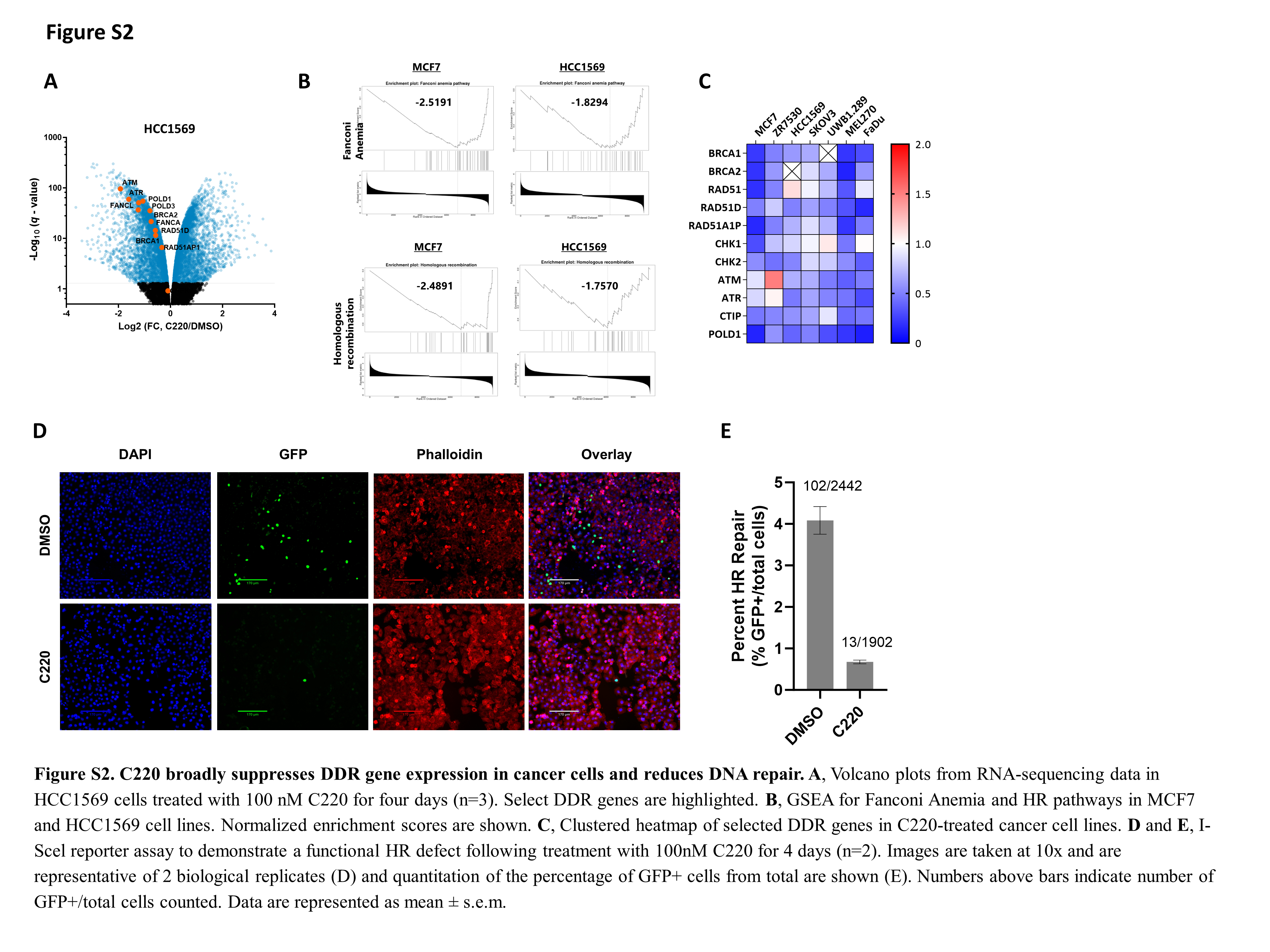

Supplement: Figure S2 — C220 broadly suppresses DDR gene expression in cancer cells and reduces DNA repair. [file crc-23-0070-s02.png]

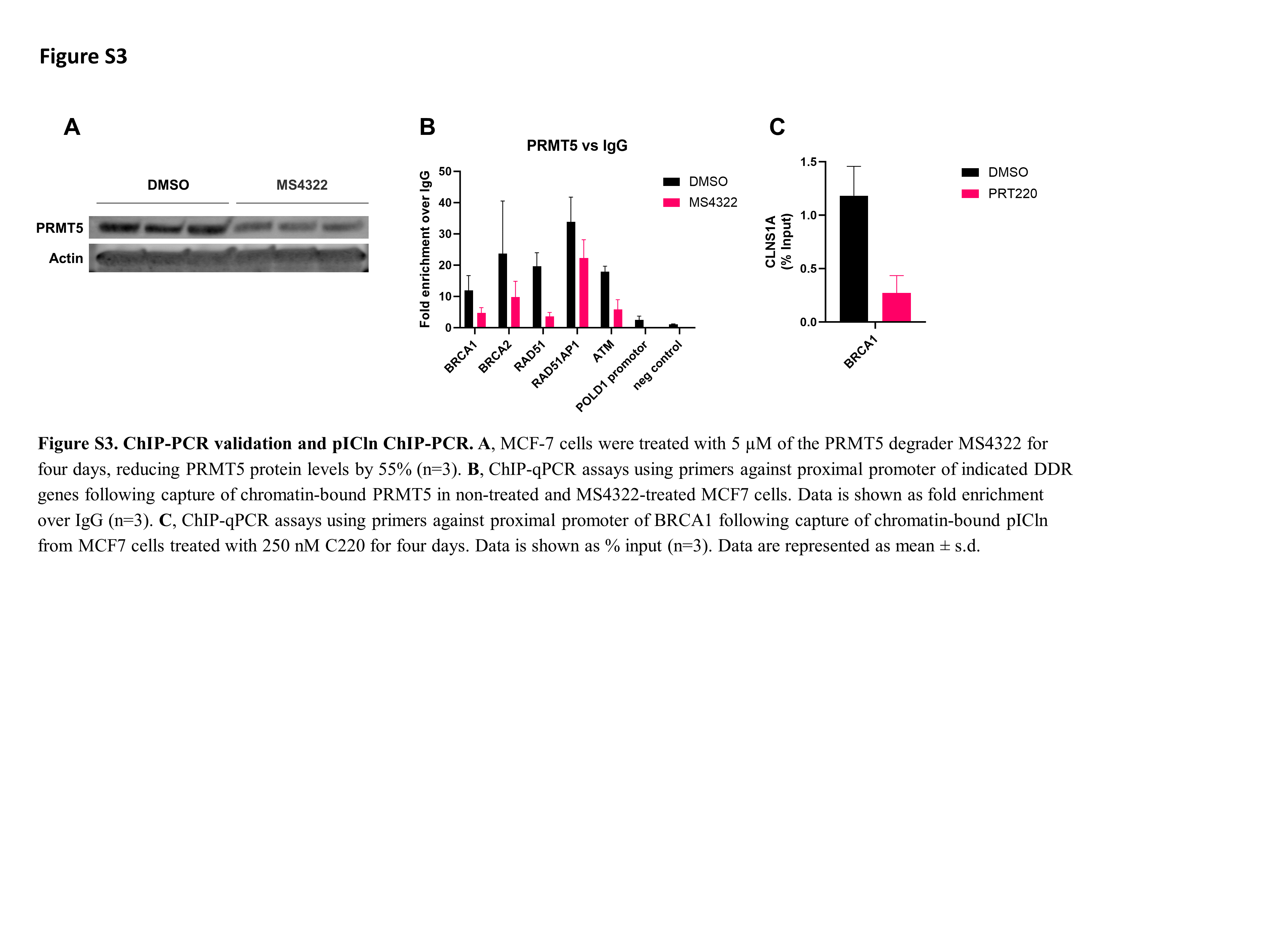

Supplement: Figure S3 — ChIP-PCR validation and pICln ChIP-PCR [file crc-23-0070-s03.png]

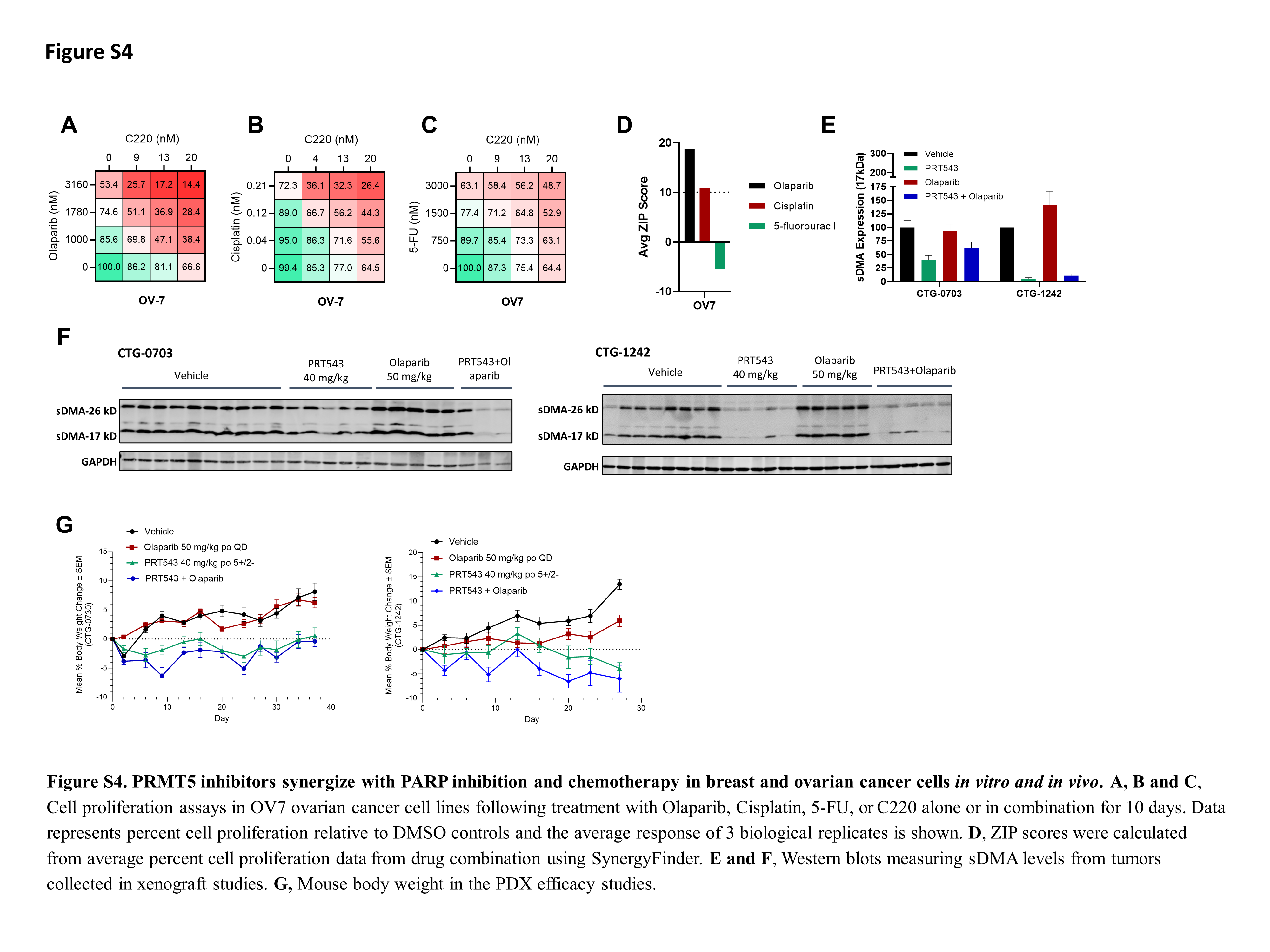

Supplement: Figure S4 — PRMT5 inhibitors synergize with PARP inhibition and chemotherapy in breast and ovarian cancer cells in vitro and in vivo. [file crc-23-0070-s04.png]

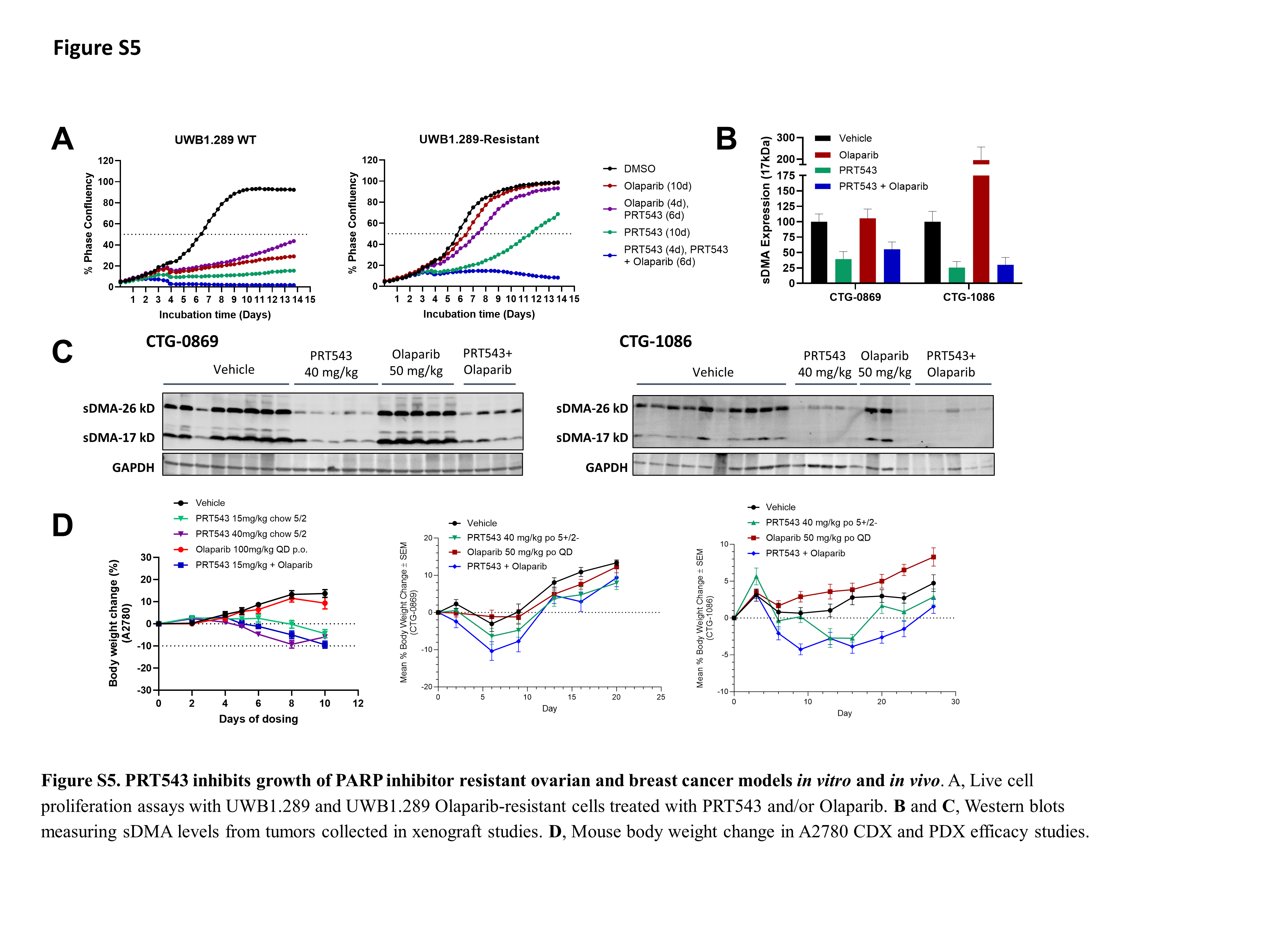

Supplement: Figure S5 — PRT543 inhibits growth of PARP inhibitor resistant ovarian and breast cancer models in vitro and in vivo. [file crc-23-0070-s05.png]
